# Systematic Evaluation of Research Progress on Natural Language Processing in Medicine Over the Past 20 Years: Bibliometric Study on PubMed

**DOI:** 10.2196/16816

**Published:** 2020-01-23

**Authors:** Jing Wang, Huan Deng, Bangtao Liu, Anbin Hu, Jun Liang, Lingye Fan, Xu Zheng, Tong Wang, Jianbo Lei

**Affiliations:** 1 School of Medical Informatics and Engineering Southwest Medical University Luzhou China; 2 IT Center, Second Affiliated Hospital School of Medicine Zhejiang University Hangzhou China; 3 Affiliated Hospital Southwest Medical University Luzhou China; 4 Center for Medical Informatics Peking University Beijing China; 5 School of Public Health Jilin University Jilin China; 6 Institute of Medical Technology Health Science Center Peking University Beijing China

**Keywords:** natural language processing, clinical, medicine, information extraction, electronic medical record

## Abstract

**Background:**

Natural language processing (NLP) is an important traditional field in computer science, but its application in medical research has faced many challenges. With the extensive digitalization of medical information globally and increasing importance of understanding and mining big data in the medical field, NLP is becoming more crucial.

**Objective:**

The goal of the research was to perform a systematic review on the use of NLP in medical research with the aim of understanding the global progress on NLP research outcomes, content, methods, and study groups involved.

**Methods:**

A systematic review was conducted using the PubMed database as a search platform. All published studies on the application of NLP in medicine (except biomedicine) during the 20 years between 1999 and 2018 were retrieved. The data obtained from these published studies were cleaned and structured. Excel (Microsoft Corp) and VOSviewer (Nees Jan van Eck and Ludo Waltman) were used to perform bibliometric analysis of publication trends, author orders, countries, institutions, collaboration relationships, research hot spots, diseases studied, and research methods.

**Results:**

A total of 3498 articles were obtained during initial screening, and 2336 articles were found to meet the study criteria after manual screening. The number of publications increased every year, with a significant growth after 2012 (number of publications ranged from 148 to a maximum of 302 annually). The United States has occupied the leading position since the inception of the field, with the largest number of articles published. The United States contributed to 63.01% (1472/2336) of all publications, followed by France (5.44%, 127/2336) and the United Kingdom (3.51%, 82/2336). The author with the largest number of articles published was Hongfang Liu (70), while Stéphane Meystre (17) and Hua Xu (33) published the largest number of articles as the first and corresponding authors. Among the first author’s affiliation institution, Columbia University published the largest number of articles, accounting for 4.54% (106/2336) of the total. Specifically, approximately one-fifth (17.68%, 413/2336) of the articles involved research on specific diseases, and the subject areas primarily focused on mental illness (16.46%, 68/413), breast cancer (5.81%, 24/413), and pneumonia (4.12%, 17/413).

**Conclusions:**

NLP is in a period of robust development in the medical field, with an average of approximately 100 publications annually. Electronic medical records were the most used research materials, but social media such as Twitter have become important research materials since 2015. Cancer (24.94%, 103/413) was the most common subject area in NLP-assisted medical research on diseases, with breast cancers (23.30%, 24/103) and lung cancers (14.56%, 15/103) accounting for the highest proportions of studies. Columbia University and the talents trained therein were the most active and prolific research forces on NLP in the medical field.

## Introduction

Natural language processing (NLP) refers to the ability of machines to understand and explain the way humans write and talk. It involves studying various theories and methods that can realize effective communication between humans and computers in natural language and is an important direction in the field of artificial intelligence [1]. The goal of NLP is to realize human-like language understanding for a wide range of applications and tasks [2]. The earliest study on natural language understanding was the machine translation design first proposed by American Warren Weaver in 1949 [3].

In modern medical care, electronic health record (EHR) and electronic medical record (EMR) systems are undergoing rapid and large-scale development [4]. For example, in 2011, the Chinese government invested ¥630 million (US $97 million) to conduct a pilot project on primary medical and health care information systems for EHR, EMR, and outpatient management [5,6]. Medical records are valuable assets of hospitals that contain a large amount of important information, such as patients’ chief complaints, diagnostic information, drugs administered, and adverse reactions. However, medical records have long been ineffectively used due to technological limitations and unstructured text formats [7]. NLP can transform these unstructured medical texts into structured data that contain important medical information from which scientists and medical personnel can identify useful medical data [8,9], thereby improving the quality and reducing the operating costs of the medical system. An increasing number of practical problems in medicine can now be solved using NLP, such as the detection of adverse drug reactions [10,11], information extraction from EHR [12], and EMR or EHR classification [13]. NLP can also be used to process issues in radiology research [14,15]. The use of NLP to aid the resolution of medical problems is advancing rapidly and drawing increasing attention [16].

With the rapid development of NLP in the medical field, there is a constant increase in the number of NLP-related articles, which has led to the accumulation of a substantial amount of research findings. Analyzing these articles can indirectly reflect the dynamic progress of NLP development in the medical field. Moreover, the results of the analysis can provide various benefits to academia, especially to scholars who are interested in pursuing careers in specific areas. Regarding the analysis and research, the studies by Cobo et al [17,18] define bibliometrics as the use of statistical methods for quantitative assessment of academic output. Bibliometrics is often used to discover top authors and institutions in a field [19], determine the structure of a research field [20], identify important topics [21], and mine research directions [22].

Previous studies have analyzed and summarized the applications of NLP in the medical field. For example, Chen et al [23] conducted a bibliometric analysis of the outcomes of NLP in medical research over 10 years from 2007 to 2016. The authors comprehensively discussed the current research status in the field, including the top authors and institutions. However, their study only analyzed 10 years of data and covered NLP research in all biomedical fields, not specifically medical research. In addition, details on the collaborative relationships between prolific authors and the diseases studied using NLP were not described. In 2015, Névéol et al [24] published a systematic review in which they focused on screening NLP methods that had been applied to clinical texts or clinical outcomes in the year of 2014 through searching bibliographic databases. In 2016, Névéol et al [25] summarized the outstanding papers on clinical NLP in the previous year. These studies mainly summarized recent research and presented a selection of the best papers published in the field of clinical NLP but lacked a comprehensive analysis of the use of NLP in the medical field.

Other previously published studies [23-26] have also summarized the role of NLP in medical research; however, they have essentially only summarized the basic characteristics, such as the number of published articles on NLP, author information, and keywords. Systematic analyses on other major features of NLP in the medical field, such as the collaboration among authors, popular research topics, and current status of the key diseases involved have not been conducted. Therefore, a systematic review spanning a longer period of time with more systematic and comprehensive analyses is necessary. This study differs from previous publications in the following aspects: first, bibliometrics was employed to review the relevant materials of medical NLP spanning nearly 20 years, which was the longest time span compared with previous studies; second, in addition to the analysis of certain basic characteristics as in previous studies, we used the VOSviewer tool version 1.6.10 (Centre for Science and Technology Studies, Leiden University) to perform cluster analyses on the relationships among authors and popular research topics. Third, we provided detailed discussion on multiple aspects of NLP, such as the diseases involved in NLP research and research tasks performed using NLP. In addition, to highlight the applications of NLP in the medical field that aligned more closely to clinical practice, we specifically excluded studies in the biomedical field, such as molecular biology, to provide more research reference materials for peers who conduct NLP research in the medical field.

## Methods

### Data Sources and Search Strategies

PubMed is an important search engine. The source of the PubMed database is MEDLINE, and the core topic is medicine. The objective of this study was to collect academic articles on the application of NLP in medicine. Therefore, PubMed was selected as the search engine in this study. On the PubMed platform, the search strategy was (“natural language processing” [all fields] OR NLP [all fields]) AND (medical [all fields] OR health [all fields] OR clinical [all fields]), automatically translated by PubMed to: ((“natural language processing” [MeSH terms] OR (“natural” [all fields] AND “language” [all fields] AND “processing” [all fields]) OR “natural language processing” [all fields]) OR NLP [all fields]) AND (medical [all fields] OR (“health” [MeSH terms] OR “health” [all fields]) OR clinical [all fields]), and the time period spanned from 1999 to 2018.

### Inclusion and Exclusion Criteria

All published studies on the application of NLP in medicine (except biomedicine) during the 20 years between 1999 and 2018 were retrieved. A total of 3498 articles were retrieved. The articles were screened according to the following exclusion criteria:

- Articles with indeterminate content were excluded, including PubMed articles without abstracts and articles with abstracts but the term NLP could not be retrieved from the abstracts and the full text could not be found.
- Review and comment articles were excluded.
- Articles with content unrelated to NLP were excluded; for example, articles wherein the term NLP did not stand for natural language processing but for terms such as neurolinguistic programming, no light perception, and ninein-like protein or NLP was only mentioned as a previous study or future study, while the main article was unrelated to NLP.
- As the subject of this study was the application of NLP in medicine and diseases, articles on molecular biomedicine, such as studies on protein-protein interactions in biomedical studies [27], were excluded.

**Figure 1:**
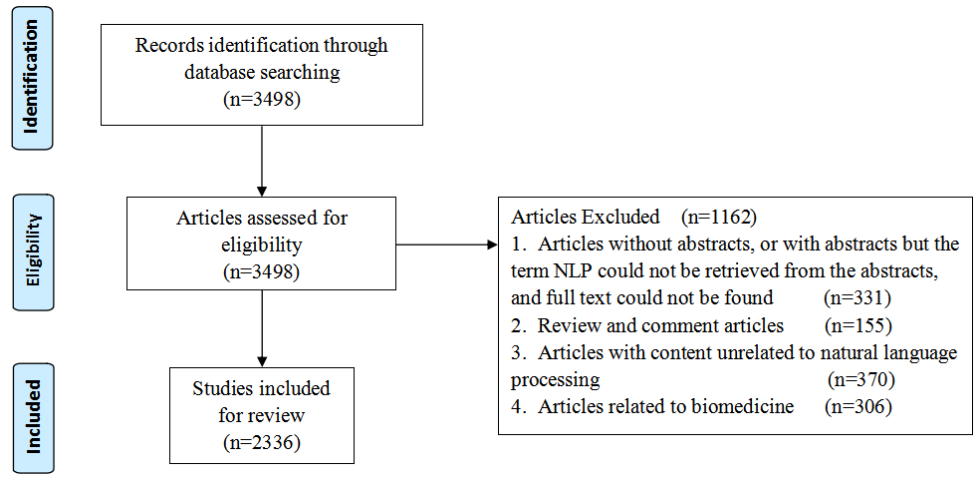
Preferred Reporting Items for Systematic Reviews and Meta-Analyses flow diagram depicting the screening procedure for articles on natural language processing (NLP) in the medical field.

The first three steps of the screening process were mainly completed by JW, and the last step of screening was jointly completed by JW and HD. In cases of discordance during the screening process on whether the article belonged to the molecular biomedical category, the two authors would review the full text and come to an agreement through discussion. We followed Preferred Reporting Items for Systematic Reviews and Meta-Analyses (PRISMA) guidelines [28], shown in Figure 1, for the screening procedure. A total of 2336 articles were included in the statistical analysis.

### Data Extraction and Statistical Analysis

The following information was extracted from eligible articles: year of publication, journal name in which the article was first published, all authors, first author, corresponding author, first author’s affiliation institution (and department), first author’s country, research tasks of NLP in the article, and disease type discussed in the article. The obtained data were input into Excel 2016 (Microsoft Corp) for data analysis and processing. Excel and VOSviewer were used in this study for the qualitative and quantitative analyses of author co-occurrences, keywords, and disease types, which helped compile and summarize the characteristics of the development of the medical NLP field in detail. The cutoff date for data collection was December 31, 2018.

## Results

### Overall Analysis of Article Data

#### Trends in Number of Articles

**Figure 2:**
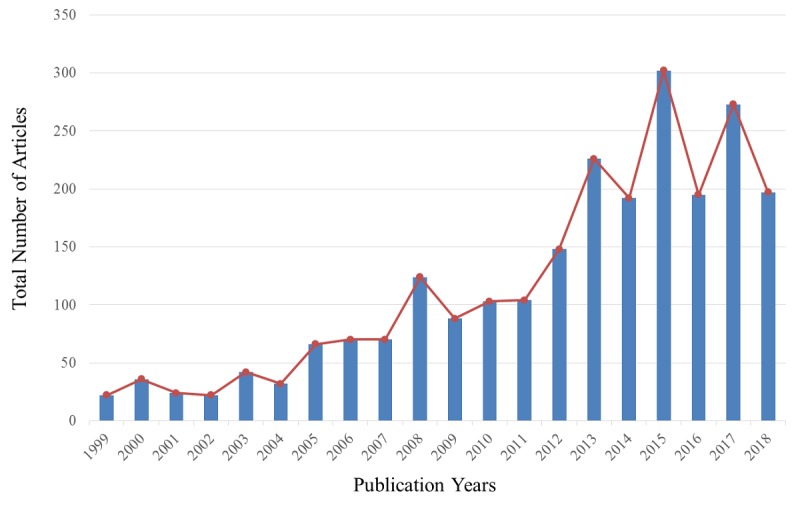
Graph showing the number of articles published over time.

Of the 2336 articles that met the study criteria, the time period spanned from 1999 to 2018. The overall trend (Figure 2) showed that the number of published articles increased every year. The time period was mainly divided into 3 phases: between 1999 and 2004 was the lag period, in which the development of the field was relatively slow, with an average of 30 (22 to 42) articles published; between 2005 and 2011 was the slow growth period, with an average of 89 (66 to 124) articles published; after 2012, NLP in the medical field entered a fast growth period. Until 2018, a yearly average of 219 (148 to 302) articles were published, with the peak (302) attained in 2015.

#### Journals in Which Articles Were Published

A total of 2336 articles were published in 412 journals. Table 1 shows the names of the top 10 journals and the corresponding number of articles in each journal. These 10 journals together contained more than 50% of the total number of articles.

**Table 1:** Medical natural language processing journal rankings (n=2336).

Rank	Journal or proceedings	Publications, n (%)
1	Studies in Health Technology and Informatics	408 (17.47)
2	AMIA Annual Symposium Proceedings	386 (16.53)
3	Journal of the American Medical Informatics Association	256 (10.96)
4	Journal of Biomedical Informatics	223 (9.55)
5	International Journal of Medical Informatics	54 (2.31)
6	BMC Medical Informatics and Decision Making	50 (2.14)
7	BMC Bioinformatics	43 (1.84)
8	AMIA Joint Summits on Translational Science Proceedings	31 (1.33)
9	Plos ONE	31 (1.33)
10	Journal of Digital Imaging	30 (1.28)

### Analysis of Author-Related Data

#### Author Orders

This study screened for the first author, corresponding author, and contributing authors of each article. The top 10 authors in each category are presented in Table 2 and Table 3. Specifically, Hongfang Liu, Hua Xu, and Joshua C Denny were ranked as the top three authors with the most number of articles published. The top three first authors were Stéphane Meystre, Özlem Uzuner, and Hua Xu, and the top three corresponding authors were Hua Xu, Stéphane Meystre and Özlem Uzuner and Carol Friedman (tie). There were four authors whose names appeared top 10 in each of the three categories: Hua Xu, Joshua C Denny, Wendy W Chapman, and Özlem Uzuner.

**Table 2:** Rank of top authors by number of articles published and the most articles published as the first plus corresponding author.

Total (first + corresponding + coauthor)			Total (first + corresponding)	
Rank	Authors	Publications	Publications	Rank
1	Hongfang Liu	70	21 (7+14)	6
2	Hua Xu	66	48 (15+33)	1
3	Joshua C Denny	64	26 (12+14)	4
4	Carol Friedman	60	20 (6+14)	7
5	Wendy W Chapman	55	25 (11+14)	5
6	Guergana Savova	45	—	—
6	Christopher G Chute	45	—	—
8	Serguei Pakhomov	43	—	—
9	Özlem Uzuner	37	—	—
9	George Hripcsak	37	—	—
9	Thomas C Rindflesch	37	—	—
—	Stéphane Meystre	—	32 (17+15)	2
—	Özlem Uzuner	—	30 (16+14)	3

**Table 3:** Top first authors and corresponding authors.

Author designation		Rank	Publications
**First**			
	Stéphane Meystre	1	17
	Özlem Uzuner	2	16
	Hua Xu	3	15
	Louise Deleger	4	13
	Joshua C Denny	5	12
	Serguei Pakhomov	5	12
	Wendy W Chapman	7	11
	Sunghwan Sohn	8	10
	Li Zhou	9	9
	Guergana Savova	9	9
**Corresponding**			
	Hua Xu	1	33
	Stéphane Meystre	2	15
	Özlem Uzuner	3	14
	Carol Friedman	3	14
	Hongfang Liu	3	14
	Wendy W Chapman	3	14
	Joshua C Denny	3	14
	Imre Solti	8	11
	Genevieve B Melton	9	10
	Hong Yu	9	10

#### Countries in Which Authors Were Based

**Figure 3:**
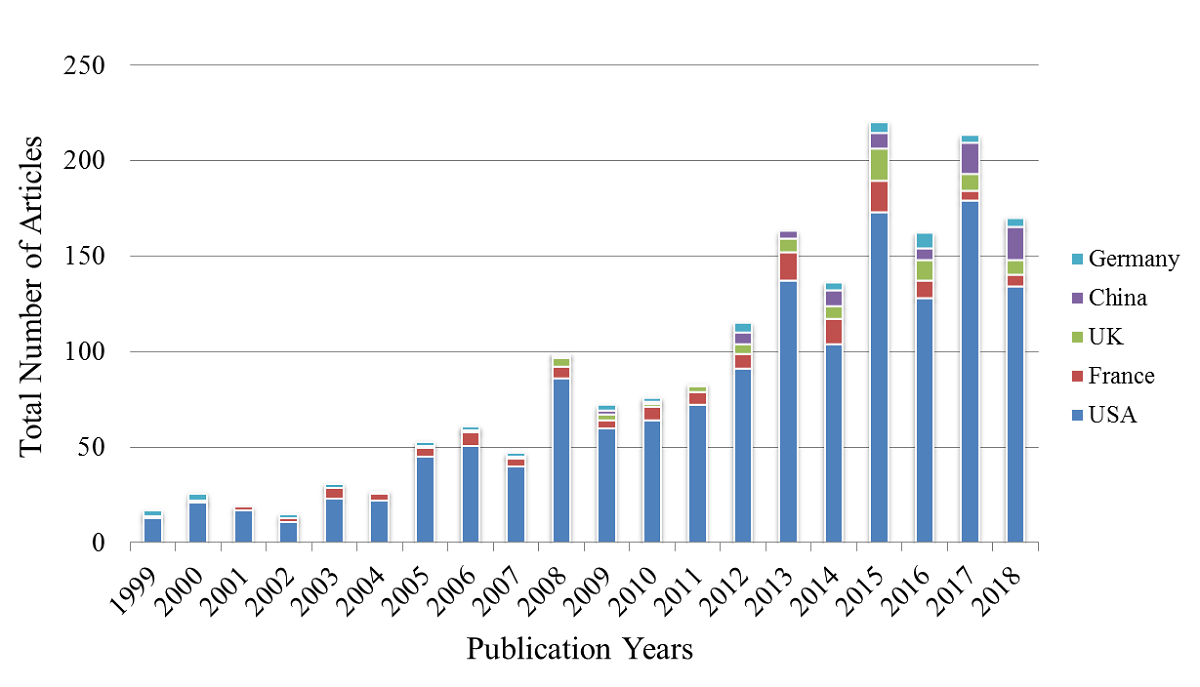
Trend in the number of articles published over 20 years in the top five countries with the most articles published.

This study first analyzed the countries in which the first authors’ institutions were located. The top 10 countries and the articles published are listed in Table 4, which shows that the United States is the top country and has contributed more than 50% of the total number of articles (63.01%), followed by France (5.44%), the United Kingdom (3.51%), and China (3.04%). Furthermore, in 2015 and 2017, the United States stood out with more than 150 articles published. Next, we analyzed the trend in the number of articles published in the top five countries over 20 years (Figure 3).

**Table 4:** Ranking of the first author’s countries (top 10, n=2336).

Rank	Country	Publications, n (%)
1	United States	1472 (63.01)
2	France	127 (5.44)
3	United Kingdom	82 (3.51)
4	China	71 (3.04)
5	Germany	57 (2.44)
6	Australia	56 (2.40)
7	Japan	52 (2.23)
8	Switzerland	44 (1.88)
9	Canada	33 (1.41)
10	Spain	28 (1.20)

#### Institutions to Which Authors Belonged

This study analyzed the relevant data on the institutions from which the articles were published. Specifically, the primary institutions to which the first authors belonged were analyzed (Table 5). The data showed that the top three institutions were Columbia University (4.54%), University of Utah (4.15%), and Mayo Clinic (3.85%). Together, these three institutions contributed a total of 12.54% of the articles published.

**Table 5:** Ranking of institutions to which the first authors belonged (n=2336).

Rank	Institution name	Publications, n (%)
1	Columbia University	106 (4.54)
2	University of Utah	97 (4.15)
3	Mayo Clinic	90 (3.85)
4	Vanderbilt University	59 (2.53)
5	National Library of Medicine	57 (2.31)
6	Brigham and Women’s Hospital	52 (2.24)
7	University of California	47 (2.01)
8	University of Pittsburgh	38 (1.63)
9	Massachusetts General Hospital	37 (1.58)
10	University of Minnesota	32 (1.37)

#### Departments to Which Authors Belonged

This study evaluated the professional background of the first authors and analyzed the departments to which the first authors belonged, with the aim of observing the overall development of NLP in the medical field across the broad range of the discipline. As statistical analysis of institutions in this study focused on the primary institutions to which the authors belonged, analysis of departments also focused on departments of the primary institutions. If an author was affiliated to multiple departments, all departments were included in the statistical analysis. Table 6 shows that the top four departments are biomedical informatics (14.3%), computer science (6.0%), radiology (3.2%), and medical informatics (2.4%).

**Table 6:** Distribution of departments to which the first authors belonged (n=2336).

Rank	Name of department	Publications, n (%)
1	Department of biomedical informatics	334 (14.30)
2	Department of computer science	141 (6.04)
3	Department of radiology	75 (3.21)
4	Department of medical informatics	55 (2.35)
5	Department of psychiatry	37 (1.58)
6	Department of neuroscience	35 (1.50)
7	Department of nursing	30 (1.28)
8	Department of health sciences	28 (1.20)
9	Department of medicine	22 (0.94)
10	Department of health informatics	19 (0.81)

#### Collaboration Status Among Authors

**Figure 4:**
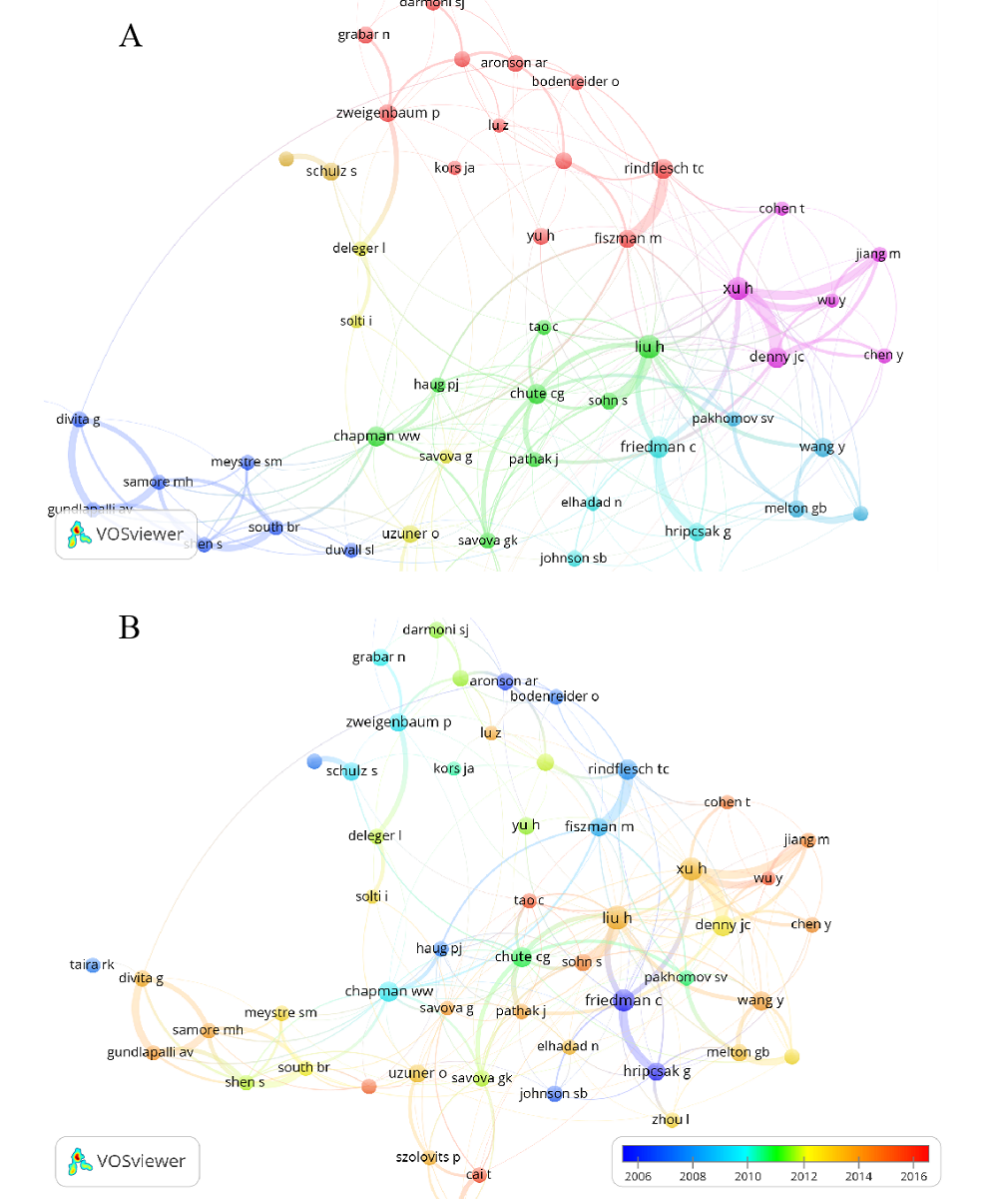
(A) Network visualization of author co-occurrences analyzed using VOSviewer. A circle represents an author, the size of the circle represents the importance, and the thickness of the link connecting the circles represents the relatedness of the connections. Circles with the same color belong to the same cluster. (B) Overlay visualization generated in VOSviewer (Centre for Science and Technology Studies, Leiden University). A color closer to blue represents an earlier time and closer to red represents a time closer to 2018 (note: refer to Multimedia Appendix 1 for details on the two diagrams and related discussions).

VOSviewer is a bibliometric analysis software for constructing and visualizing bibliometric maps. It was codeveloped by Nees Jan van Eck and Ludo Waltman of Leiden University in the Netherlands [29], and it has unique advantages in clustering techniques based on co-occurrences. VOSviewer provides three types of map visualizations: network visualization, overlay visualization, and density visualization. VOSviewer was used in this study to analyze the collaboration status among authors, and the network visualization and overlay visualization of VOSviewer were employed. The network visualization could provide clusters of top authors in the field. This, together with the overlay visualization, could provide the distribution of timing of collaboration in each author cluster to understand their collaboration trends. The directions of collaboration and research objectives of each author cluster could then be obtained through reviewing the corresponding articles. When performing analysis using VOSviewer in this study, the minimum number of documents of an author was set to 20. As shown in Figure 4A, the article authors were divided into six large clusters, and Figure 4B shows the distribution of collaboration time among the authors.

### Keyword Analysis

**Figure 5:**
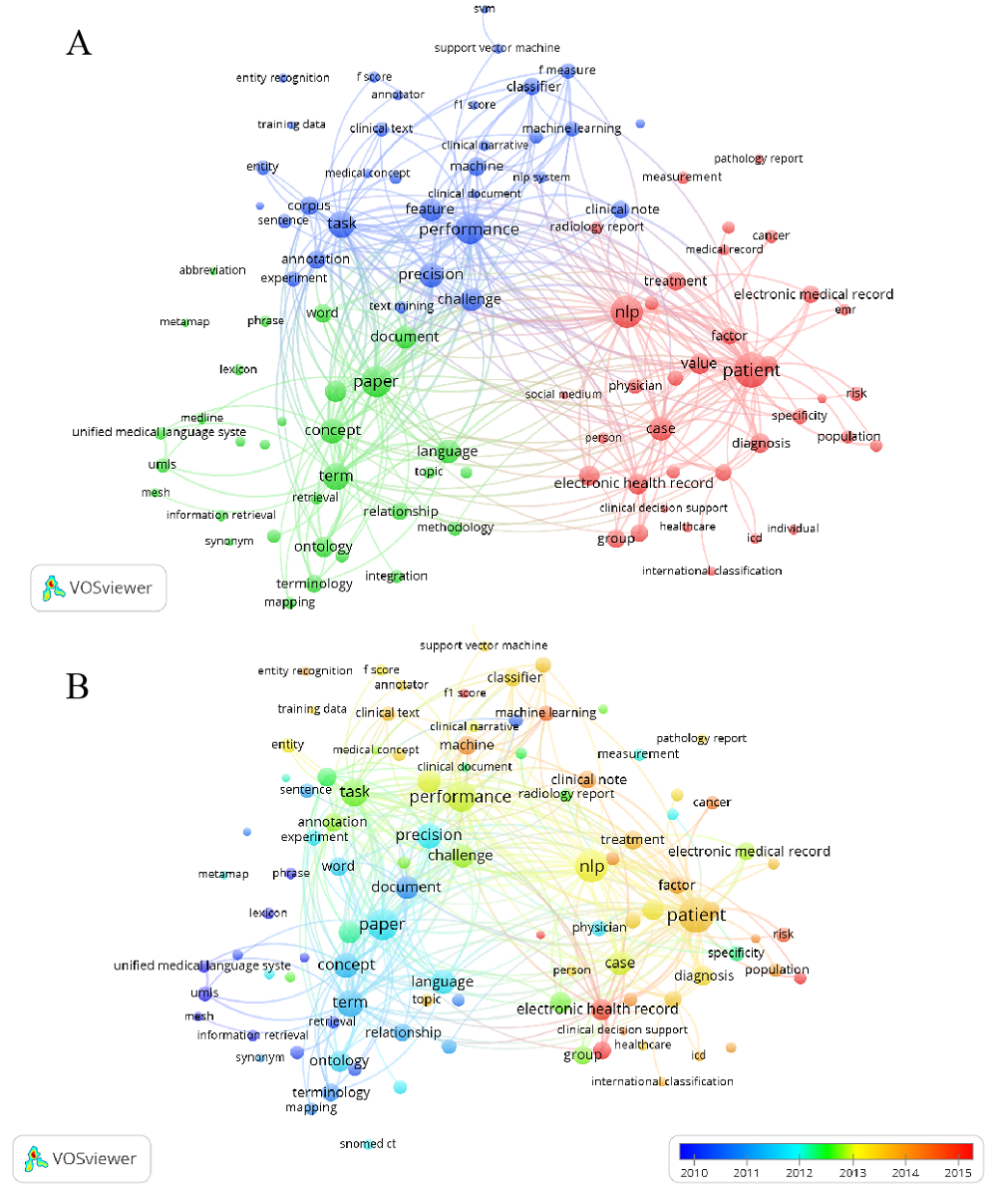
(A) Distribution of keywords. A circle represents an identified keyword, the size of the circle represents the importance, and the thickness of the link connecting the circles represents the relatedness of the connections among the keywords. Circles with the same color belong to the same cluster. (B) Changes in keywords over time. A color closer to blue represents an earlier time and closer to red represents a time closer to 2018 (note: refer to Multimedia Appendix 1 for details on the two diagrams and related discussions).

Analysis of keywords can indirectly reveal hotspots and changing trends in research topics, critical for understanding the development of this field [30]. VOSviewer was used in this study to perform keyword analysis. The purpose of the analysis was to identify the most popular research hotspots in the field and obtain the changing trends in keywords over time through the overlay visualization generated in VOSviewer. This could help researchers determine potential future research directions. During statistical analysis, keywords were defined as words that were used more than 50 times in titles and abstracts in all publications. As shown in Figure 5A, 327 keywords were identified, and the keywords were grouped as red, yellow, and blue. Based on these three categories, the relatedness among these keywords can be observed. For example, in the red category, patient (978 times), electronic health record (610 times), and electronic medical record (361 times) belong to the clinical NLP field; in the blue category, classifier (249 times), machine learning (215 times), support vector machine (164 times), and information extraction (150 times) belong to NLP research methods; and in the green category, language (449 times), phrase and word (395 times), ontology (345 times), terminology (267 times), and lexicon (106 times) belong to NLP research subjects. Next, the overlay visualization (Figure 5B) shows the trends in keyword changes as time progresses. In Figure 5B, blue indicates that the timing of appearance is earlier, and red indicates that the timing of appearance is later. The figure reveals certain hotspots have developed in the field in recent years, including electronic health record (176 times in 2014), cancer (19 times in 2014), and machine learning (34 times in 2014). It is worth noting that social media in the red category appeared 22 times in 2016.

### Analysis of Current Status of Specific Diseases Studied Using Natural Language Processing

This study found that 413 articles mentioned specific diseases studied using NLP, accounting for about one-fifth of the total number of articles. We conducted a comprehensive analysis of these articles to understand the type of disease information mined by NLP and how it was performed. This could provide a reference tool for the use of NLP when studying disease cases in the future.

#### Current Status of Specific Diseases Studied Using Natural Language Processing

**Figure 6:**
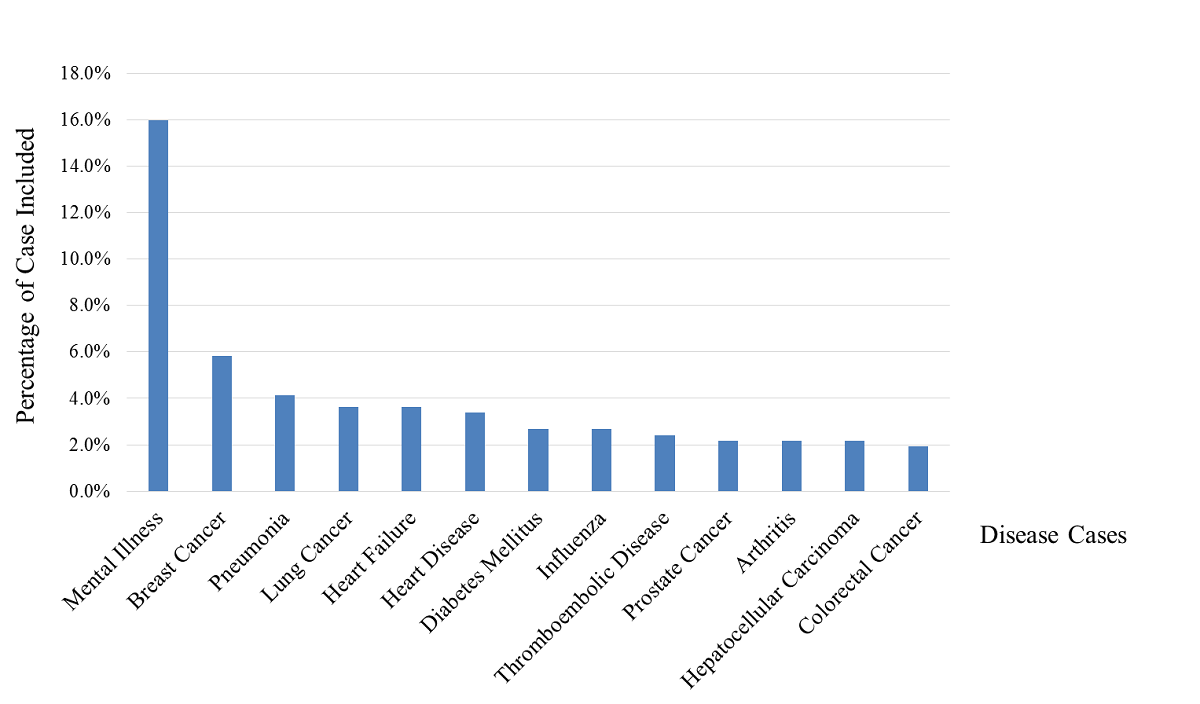
Ranking of disease categories based on studies that used natural language processing for the investigation of disease cases.

Of the 413 articles, the categories of diseases studied using NLP are shown in Figure 6. Specifically, mental illness ranked at the top, accounting for 16.5% (68/413) of the articles. The second and third ranks were breast cancer (5.8%, 24/413) and pneumonia (4.1%, 17/413). The names of the diseases in the Figure 6 were mainly based on the specific disease names mentioned in the article.

#### Specific Diseases Studied Using Natural Language Processing by Time Period

**Figure 7:**
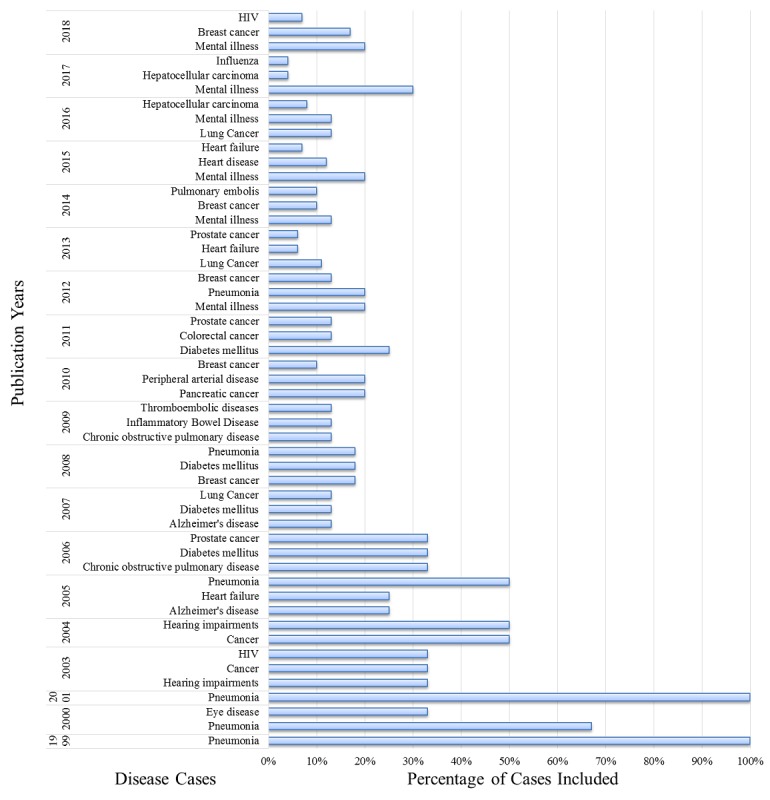
Temporal distribution of studies that used natural language processing for the investigation of disease cases (note: this figure shows the names of the top three diseases in studies that used natural language processing to investigate disease cases each year. Fewer than three disease types indicates that only one or two diseases were studied in the year. The term cancer in the figure indicates the article only mentioned the term cancer, without specifying the type of cancer).

The temporal distribution of NLP research used to study diseases was analyzed in this study. As shown in Figure 7, initially in 1999, only one article clearly stated the type of disease that involved the use of NLP: pneumonia. In the next 3 years, pneumonia remained the main subject area in NLP research. From 2006, the use of NLP for the study of cancer cases had become popular, with a primary focus on lung cancer, prostate cancer, and breast cancer. The use of NLP in breast cancer research was mainly concentrated in 2018, with 10 articles published, almost all of them were from the United States. In addition, diseases such as diabetes, mental illness, and prostate cancer were all common subject areas in NLP research.

#### Current Status of Diseases Studied Using Natural Language Processing by Country

**Figure 8:**
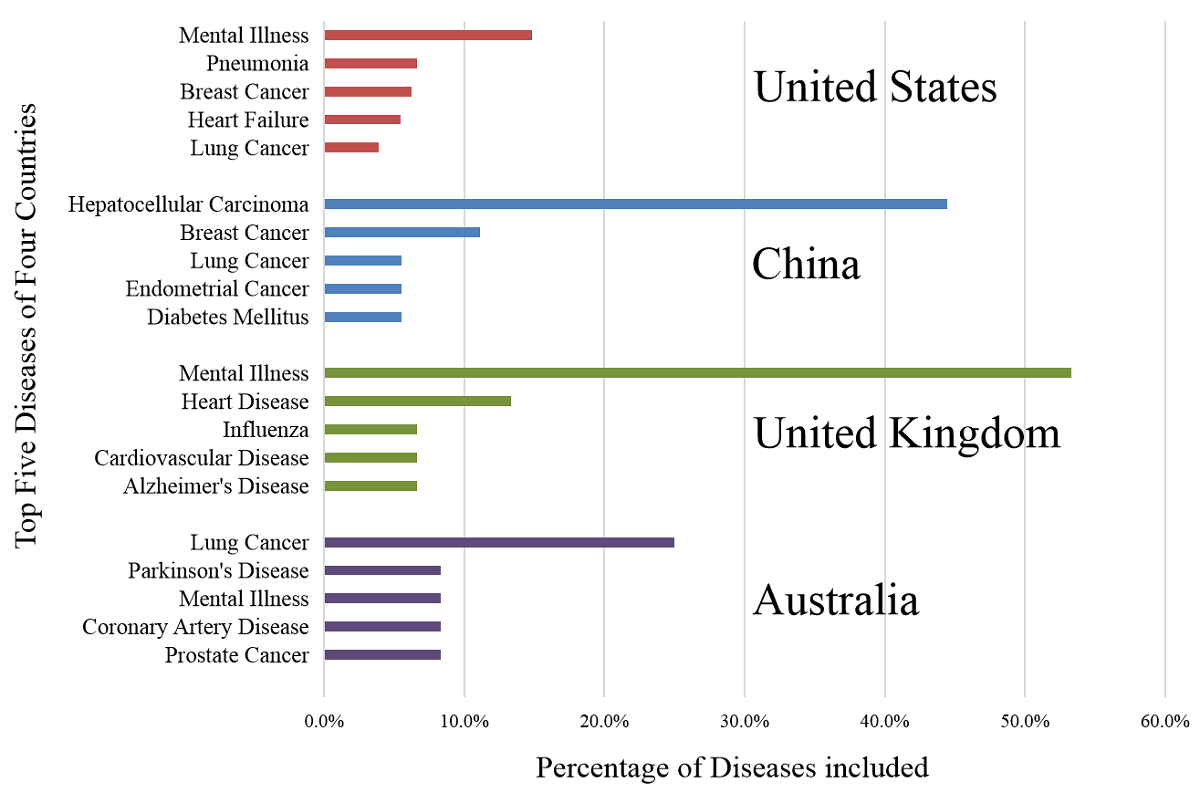
Distribution of diseases in studies that used natural language processing for the investigation of disease cases in the United States, China, United Kingdom, and Australia.

Of the 413 articles that studied disease cases using NLP, the top four countries from where the first authors were located were the United States (68.3%, 282/413), China (4.8%, 20/413), the United Kingdom (3.6%, 15/413), and Australia (3.1%, 13/413). This ranking was consistent with the total number of articles published by country. The status of NLP research for use to study disease cases in these four countries was further investigated. As shown in Figure 8, the research subjects in the United States were more diverse, and there was no specific area of focus. The key subject area studied in China was hepatocellular carcinoma. The United Kingdom and Australia mainly focused on mental illness and lung cancer.

### Research Tasks of Natural Language Processing in the Medical Field

The abstracts of 2336 articles were analyzed in this study to explore the research tasks of NLP involved in each article. If the abstract did not mention the specific task of NLP, the full text was reviewed. If the task could not be clearly identified from the full text, the article would be excluded from the analysis. NLP tasks involved were undetermined in 73 articles.

**Figure 9:**
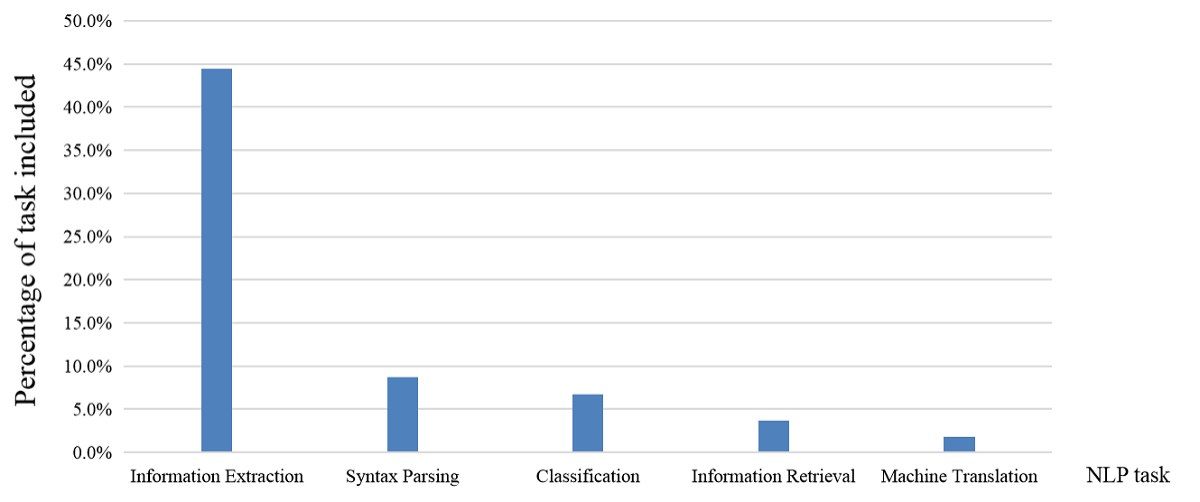
Top five ranks of the research tasks of natural language processing (NLP) in the medical field.

The authors of this study referenced the content on NLP described in chapter 4 of *Artificial Intelligence and its Application, Fourth Edition* [31], and divided the NLP tasks into speech recognition, machine translation, syntax parsing, classification, information retrieval, information extraction, information filtering, natural language generation, sentiment analysis, question answering system, and so on. This study analyzed the number of articles related to each NLP task and found that the top five tasks were information extraction (44.41%, 1005/2263), syntax parsing (8.66%, 196/2263), classification (6.72%, 152/2263), information retrieval (3.71%, 84/2263), and machine translation (1.77%, 40/2263; Figure 9).

## Discussion

### Overall Development Status of Medical Natural Language Processing

NLP research in the past 20 years could be divided into 3 phases: the lag period (1999-2004) with a yearly average of 30 (22 to 42) articles published, the slow growth period (2005-2011) with a yearly average of 89 (66 to 124) articles published, and the fast growth period (2012-2018) with a yearly average of 219 articles (148 to 302) articles published, with a peak (302) attained in 2015. Analysis by country showed that the United States has been the leader since the beginning of NLP development. Prior to 2008, only the United States, France, and Germany, with few exceptions, had conducted investigations in the field. Of the five countries shown in Figure 3, China started the latest and only began to emerge in the field in 2012. The development of NLP in Germany has remained relatively stable without a particular outstanding year, and Germany generally ranked in the fourth or fifth position. The development of NLP in France has also been relatively stable. In the first 15 years, France usually occupied the second position, but it has been surpassed by China in the past 2 years. Between 2016 and 2018, China has published nearly 40 articles, with a primary focus on hepatocellular carcinoma research assisted by NLP, as well as the use of NLP to mine or identify relevant information in clinical notes or EMR.

### Analysis of Prolific Authors and Affiliation Institutions

This study identified the prominent authors who had made significant contributions to the NLP field, and we noted the following salient feature: the top two authors with the highest number of publications, Hongfang Liu and Hua Xu, plus Carol Friedman (ranked fourth rather than first because quite a few of her articles are about methodology and biology, which were not included in the scope of this study, but this does not change that she is recognized as a leading pioneer in this field) and George Hripcsak, ninth position, were all from Columbia University. In particular, Carol Friedman and George Hripcsak are currently at Columbia University, whereas Hongfang Liu and Hua Xu are both students of Carol Friedman. Among the top five prolific authors who published as the first plus corresponding author, Hua Xu (ranked first), Hongfang Liu (ranked sixth), and Carol Friedman (ranked seventh), were all from Columbia University. In addition, analysis of the first author’s affiliation institutions showed that Columbia University (106) was ahead of University of Utah (97) in second place and the Mayo Clinic (90) in third place. These findings indicated that Columbia University and its students were the most active in the field of medical NLP research.

Notably, as shown in Table 3, the top 10 institutions to which the first authors belonged were all from the United States, including 6 universities, 3 hospitals, and 1 library. This also reflects that universities are the key locations for conducting medical NLP research.

Analysis by department showed that the top four majors were biomedical informatics, computer science, radiology, and medical informatics. These four majors mainly involve the processing of highly integrated data using computers and the expertise involved related to interdisciplinary content, such as medical information. It was evident that researchers with professional backgrounds in these fields had contributed significantly to the development of NLP. The research and study of NLP should be the key learning direction for future students majoring these subjects.

### Current Development Status of Natural Language Processing Research on Disease Investigations

Analysis of this study showed that the top disease type in disease research involving NLP was mental illness. The World Health Organization predicts that mental illness may become the third most common human disease in the world in the future, after heart disease and cancer [32], showing the severity of the risk posed by this illness. NLP plays an indispensable role in mental illness research. For example, Victor et al [33] used NLP to train a diagnostic algorithm with 95% specificity for classifying bipolar disorder. It has been shown that NLP of EHRs is increasingly being used to study mental illness [34].

The journal Lancet Oncology published global cancer statistics for young people aged 20 to 39 years in 2017: one million young people in the world are diagnosed with cancer each year, and breast cancer is the most commonly diagnosed cancer (20%) [35]. Faced with such severe circumstances, Zeng et al [36] used NLP to investigate challenging issues in breast cancer such as local recurrence.

From 1999 to 2005, NLP was often used to study pneumonia cases. Our analysis showed that the main role of NLP in studies on pneumonia cases was the identification of pneumonia-related concepts from chest radiograph reports, or the use of NLP to complete automatic coding of pneumonia-related concepts. In addition, Jones et al [37] used a natural language processing tool to identify patients for pneumonia across US Department of Veterans Affairs emergency departments. The additional assistance provided by NLP improved physicians’ ability to identify pneumonia and facilitated clinical decision making by physicians.

Among disease research involving NLP, China ranked second regarding the number of articles published (20 articles). Figure 8 shows that half the studies conducted by Chinese researchers exploring diseases using NLP are on hepatocellular carcinoma. Hepatocellular carcinoma is a primary liver cancer with a high mortality rate. Research on hepatocellular carcinoma in China was concentrated in 2016 and 2017. The research direction was mainly in two areas: (1) information extraction using NLP for mining relevant data [38] and (2) combining NLP analysis with other analyses, such as pathway analysis and ontology analysis, to mine the role of related genes in hepatocellular carcinoma, such as microRNA-132 and microRNA-223-3p [39].

### Research Tasks of Natural Language Processing in Medicine

According to the results of this study, and as shown in Figure 9, the most widely performed tasks by NLP in the medical field were information extraction, syntax parsing, classification, information retrieval, and machine translation. We will now discuss these five tasks in detail.

Information extraction accounted for the highest proportion of all medical NLP tasks. Almost one-third of medical NLP tasks were information extraction, indicating its importance in NLP. Information extraction mainly refers to the use of computers to automatically extract a specific type of information (such as entities, relationships, and events) from a vast number of structured or semistructured texts and to form structured data [40]. The analysis in this study, together with a previously published report [40], concludes that the development of information extraction in the medical field includes four main parts: (1) entity recognition, in which the task is to identify content such as a person’s name, time, and place from the texts and add the corresponding labeling information [41-44]; (2) anaphora resolution, which mainly refers to the way of simplifying and standardizing the expression of entities that can greatly improve the accuracy of the results from information extraction [45]; (3) relationship extraction, which obtains the grammatical or semantic connections among entities in the texts, such as temporal relationships and is a crucial element in information extraction [46,47]; and (4) event extraction, which mainly focuses on how to extract events of interest from unstructured texts containing event information and present the events expressed in natural language in a structured form [48-50]. The paper found that the platform of information extraction has gradually moved to social media; 20% of the articles obtained data through the Twitter platform [51-55].

Text classification, which is a process of automated text classification based on text content and the use of computers to automatically classify texts under a given classification system and classification criteria [31]. There were many cases involved text classification [56-58], for example, Morioka et al [56] developed a feature vector to classify the radiology reports with a decision table classifier.

Syntactic analysis, also known as parsing in natural language, uses syntax and other relevant knowledge of natural languages to determine the functions of each component that constitutes an input sentence. This technology is used to establish a data structure and acquire the meaning of the input sentence [31]. The process includes lexical analysis [59], grammatical analysis, and semantic analysis.

Information retrieval refers to the query methods and processes for searching related documents required by users from an enormous number of documents using computer systems [31]. For example, Tang et al [60] investigated a novel deep learning–based method to retrieve the similar patient question in Chinese.

Machine translation refers to the automated translation of words or speech from one natural language to another natural language using computer programs. To put in simple terms, machine translation is the conversion of words from one natural language into words of another language. More complex translations can be automated using corpora [31]. For example, Merabti et al [61] translated the Foundational Model of Anatomy terms into French using methods lexically based on several NLP tools.

### Conclusions

In this study, we conducted a bibliometric analysis and presented the development of NLP in the medical field over the past 20 years. While the United States continues to be the leader in the field, many countries such as China and the United Kingdom are also advancing rapidly. In recent years, the use of NLP has become popular to process information obtained from social media platforms—for example, studies have obtained information related to diseases and patient care from the Twitter platform. Cancer has always been one of the greatest threats to human health. The use of NLP to assist cancer research has become a recent trend, for example, for use in breast cancer and prostate cancer research. Tasks such as information extraction and syntax parsing have always been popular tasks in the medical NLP field. Future studies will focus on how to better integrate these tasks into medical NLP research.

## Appendix

Multimedia Appendix 1 Network diagrams and analysis of keywords and collaboration among authors.

## Abbreviations

EHR: electronic health record
EMR: electronic medical record
NLP: natural language processing
PRISMA: Preferred Reporting Items for Systematic Reviews and Meta-Analyses

## References

[1] Cambria E, White B (2014). Jumping NLP curves: a review of natural language processing research [review article]. IEEE Comput Intell Mag.

[2] Liddy E (2001). Natural language processing. Scripting Intelligence.

[3] Weaver W, Locke WN, Booth AD (1955). Translation. Machine Translation of Languages.

[4] Dobrow MJ, Bytautas JP, Tharmalingam S, Hagens S (2019). Interoperable electronic health records and health information exchanges: systematic review. JMIR Med Inform.

[5] Deng H, Wang J, Liu X, Liu B, Lei J (2018). Evaluating the outcomes of medical informatics development as a discipline in China: a publication perspective. Comput Methods Programs Biomed.

[6] Li Y, Hu J (2012). Health informationization of China: status and development. Chin J Health Inform Manag.

[7] Gillum RF (2013). From papyrus to the electronic tablet: a brief history of the clinical medical record with lessons for the digital age. Am J Med.

[8] Gonzalez-Hernandez G, Sarker A, O'Connor K, Savova G (2017). Capturing the patient's perspective: a review of advances in natural language processing of health-related text. Yearb Med Inform.

[9] Jung KY, Kim T, Jung J, Lee J, Choi JS, Mira K, Chang DK, Cha WC (2018). The effectiveness of near-field communication integrated with a mobile electronic medical record system: emergency department simulation study. JMIR Mhealth Uhealth.

[10] Bousquet C, Dahamna B, Guillemin-Lanne S, Darmoni SJ, Faviez C, Huot C, Katsahian S, Leroux V, Pereira S, Richard C, Schück S, Souvignet J, Lillo-Le LA, Texier N (2017). The adverse drug reactions from patient reports in social media project: five major challenges to overcome to operationalize analysis and efficiently support pharmacovigilance process. JMIR Res Protoc.

[11] Kusch MKP, Zien A, Hachenberg C, Haefeli WE, Seidling HM (2019). Information on adverse drug reactions-proof of principle for a structured database that allows customization of drug information. Int J Med Inform.

[12] Li F, Liu W, Yu H (2018). Extraction of information related to adverse drug events from electronic health record notes: design of an end-to-end model based on deep learning. JMIR Med Inform.

[13] Guo H, Na X, Hou L, Li J (2017). Classifying Chinese questions related to health care posted by consumers via the internet. J Med Internet Res.

[14] Cai T, Giannopoulos AA, Yu S, Kelil T, Ripley B, Kumamaru KK, Rybicki FJ, Mitsouras D (2016). Natural language processing technologies in radiology research and clinical applications. Radiographics.

[15] Pons E, Braun LMM, Hunink MGM, Kors JA (2016). Natural language processing in radiology: a systematic review. Radiology.

[16] Névéol A, Zweigenbaum P (2016). Clinical natural language processing in 2015: leveraging the variety of texts of clinical interest. Yearb Med Inform.

[17] Cobo M, Martínez M, Gutiérrez-Salcedo M, Fujita H, Herrera-Viedma E (2015). 25 years at knowledge-based systems: a bibliometric analysis. Knowl-Based Syst.

[18] Cobo M, López-Herrera A, Herrera-Viedma E, Herrera F (2011). An approach for detecting, quantifying, and visualizing the evolution of a research field: a practical application to the Fuzzy Sets Theory field. J Informetrics.

[19] Chen X, Chen B (2017). Discovering the recent research in natural language processing field based on a statistical approach. Lect Notes Comput Sci.

[20] Wallace ML, Larivière V, Gingras Y (2012). A small world of citations? The influence of collaboration networks on citation practices. PLoS One.

[21] Chen X, Weng H (2017). A data-driven approach for discovering the recent research status of diabetes in China. Lect Notes Comput Sci.

[22] Boudry C, Mouriaux F (2015). Eye neoplasms research: a bibliometric analysis from 1966 to 2012. Eur J Ophthalmol.

[23] Chen X, Xie H, Wang FL, Liu Z, Xu J, Hao T (2018). A bibliometric analysis of natural language processing in medical research. BMC Med Inform Decis Mak.

[24] Névéol A, Zweigenbaum P (2015). Clinical natural language processing in 2014: foundational methods supporting efficient healthcare. Yearb Med Inform.

[25] Névéol A, Zweigenbaum P (2016). Clinical natural language processing in 2015: leveraging the variety of texts of clinical interest. Yearb Med Inform.

[26] Névéol A, Zweigenbaum P (2017). Making sense of big textual data for health care: findings from the section on clinical natural language processing. Yearb Med Inform.

[27] Li L, Zhang P, Zheng T, Zhang H, Jiang Z, Huang D (2014). Integrating semantic information into multiple kernels for protein-protein interaction extraction from biomedical literatures. PLoS One.

[28] Moher D, Liberati A, Tetzlaff J, Altman DG (2009). Preferred reporting items for systematic reviews and meta-analyses: the PRISMA statement. PLoS Med.

[29] Van Eck NJ, Waltman L (2009). How to normalize cooccurrence data? An analysis of some well-known similarity measures. J Am Soc Inf Sci.

[30] Li T, Ho Y, Li C (2008). Bibliometric analysis on global Parkinson's disease research trends during 1991-2006. Neurosci Lett.

[31] Gong Z, Xu G (2015). Artificial Intelligence and its Applications.

[32] (2019). China mental health survey results summit.

[33] Castro VM, Minnier J, Murphy SN, Kohane I, Churchill SE, Gainer V, Cai T, Hoffnagle AG, Dai Y, Block S, Weill SR, Nadal-Vicens M, Pollastri AR, Rosenquist JN, Goryachev S, Ongur D, Sklar P, Perlis RH, Smoller JW, International Cohort Collection for Bipolar Disorder Consortium (2015). Validation of electronic health record phenotyping of bipolar disorder cases and controls. Am J Psychiatry.

[34] Perera G, Broadbent M, Callard F, Chang C, Downs J, Dutta R, Fernandes A, Hayes RD, Henderson M, Jackson R, Jewell A, Kadra G, Little R, Pritchard M, Shetty H, Tulloch A, Stewart R (2016). Cohort profile of the South London and Maudsley NHS Foundation Trust Biomedical Research Centre (SLaM BRC) Case Register: current status and recent enhancement of an Electronic Mental Health Record-derived data resource. BMJ Open.

[35] Fidler MM, Gupta S, Soerjomataram I, Ferlay J, Steliarova-Foucher E, Bray F (2017). Cancer incidence and mortality among young adults aged 20-39 years worldwide in 2012: a population-based study. Lancet Oncol.

[36] Zeng Z, Espino S, Roy A, Li X, Khan SA, Clare SE, Jiang X, Neapolitan R, Luo Y (2018). Using natural language processing and machine learning to identify breast cancer local recurrence. BMC Bioinformatics.

[37] Jones BE, South BR, Shao Y, Lu CC, Leng J, Sauer BC, Gundlapalli AV, Samore MH, Zeng Q (2018). Development and validation of a natural language processing tool to identify patients treated for pneumonia across va emergency departments. Appl Clin Inform.

[38] Zhang X, Tang W, Chen G, Ren F, Liang H, Dang Y, Rong M (2016). An encapsulation of gene signatures for hepatocellular carcinoma, MicroRNA-132 predicted target genes and the corresponding overlaps. PLoS One.

[39] Zhang R, Zhang L, Yang M, Huang L, Chen G, Feng Z (2018). Potential role of microRNA‑223‑3p in the tumorigenesis of hepatocellular carcinoma: a comprehensive study based on data mining and bioinformatics. Mol Med Rep.

[40] Guo X, He T (2015). Survey about research on information extraction. Comput Sci.

[41] Lei J, Tang B, Lu X, Gao K, Jiang M, Xu H (2014). A comprehensive study of named entity recognition in Chinese clinical text. J Am Med Inform Assoc.

[42] Urbain J (2015). Mining heart disease risk factors in clinical text with named entity recognition and distributional semantic models. J Biomed Inform.

[43] Han J, Chen K, Fang L, Zhang S, Wang F, Ma H, Zhao L, Liu S (2019). Improving the efficacy of the data entry process for clinical research with a natural language processing-driven medical information extraction system: quantitative field research. JMIR Med Inform.

[44] Wang SY, Pershing S, Tran E, Hernandez-Boussard T (2019). Automated extraction of ophthalmic surgery outcomes from the electronic health record. Int J Med Inform.

[45] Ware H, Mullett CJ, Jagannathan V, El-Rawas O (2012). Machine learning-based coreference resolution of concepts in clinical documents. J Am Med Inform Assoc.

[46] Foufi V, Timakum T, Gaudet-Blavignac C, Lovis C, Song M (2019). Mining of textual health information from Reddit: analysis of chronic diseases with extracted entities and their relations. J Med Internet Res.

[47] Doing-Harris K, Livnat Y, Meystre S (2015). Automated concept and relationship extraction for the semi-automated ontology management (SEAM) system. J Biomed Semantics.

[48] Karystianis G, Adily A, Schofield P, Knight L, Galdon C, Greenberg D, Jorm L, Nenadic G, Butler T (2019). Correction: automatic extraction of mental health disorders from domestic violence police narratives: text mining study. J Med Internet Res.

[49] Doryab A, Villalba DK, Chikersal P, Dutcher JM, Tumminia M, Liu X, Cohen S, Creswell K, Mankoff J, Creswell JD, Dey AK (2019). Identifying behavioral phenotypes of loneliness and social isolation with passive sensing: statistical analysis, data mining and machine learning of smartphone and fitbit data. JMIR Mhealth Uhealth.

[50] Usama M, Ahmad B, Xiao W, Hossain MS, Muhammad G (2019). Self-attention based recurrent convolutional neural network for disease prediction using healthcare data. Comput Methods Programs Biomed.

[51] Kagashe I, Yan Z, Suheryani I (2017). Enhancing seasonal influenza surveillance: topic analysis of widely used medicinal drugs using twitter data. J Med Internet Res.

[52] Pérez-Pérez M, Pérez-Rodríguez G, Fdez-Riverola F, Lourenço A (2019). Using twitter to understand the human bowel disease community: exploratory analysis of key topics. J Med Internet Res.

[53] Albalawi Y, Nikolov NS, Buckley J (2019). Trustworthy health-related tweets on social media in Saudi Arabia: tweet metadata analysis. J Med Internet Res.

[54] Garcia-Rudolph A, Laxe S, Saurí J, Bernabeu Guitart M (2019). Stroke survivors on twitter: sentiment and topic analysis from a gender perspective. J Med Internet Res.

[55] Leis A, Ronzano F, Mayer MA, Furlong LI, Sanz F (2019). Detecting signs of depression in tweets in Spanish: behavioral and linguistic analysis. J Med Internet Res.

[56] Morioka C, Meng F, Taira R, Sayre J, Zimmerman P, Ishimitsu D, Huang J, Shen L, El-Saden S (2016). Automatic classification of ultrasound screening examinations of the abdominal aorta. J Digit Imaging.

[57] Amorim P, Moraes T, Fazanaro D, Silva J, Pedrini H (2018). Shearlet and contourlet transforms for analysis of electrocardiogram signals. Comput Methods Programs Biomed.

[58] Young IJB, Luz S, Lone N (2019). A systematic review of natural language processing for classification tasks in the field of incident reporting and adverse event analysis. Int J Med Inform.

[59] Kloehn N, Leroy G, Kauchak D, Gu Y, Colina S, Yuan NP, Revere D (2018). Improving consumer understanding of medical text: development and validation of a new subsimplify algorithm to automatically generate term explanations in English and Spanish. J Med Internet Res.

[60] Tang GY, Ni Y, Xie GT, Fan XL, Shi YL (2017). A deep learning-based method for similar patient question retrieval in Chinese. Stud Health Technol Inform.

[61] Merabti T, Soualmia LF, Grosjean J, Palombi O, Müller J, Darmoni SJ (2011). Translating the foundational model of anatomy into French using knowledge-based and lexical methods. BMC Med Inform Decis Mak.

