# Supplementary material for: Systematic Evaluation of Research Progress on Natural Language Processing in Medicine Over the Past 20 Years: Bibliometric Study on PubMed
Source: J Med Internet Res. 2020 Jan 23;22(1):e16816. doi: 10.2196/16816 (PMC7005695; doi:10.2196/16816)
Supplement: Multimedia Appendix 1 [file jmir_v22i1e16816_app1.docx]

# Result

## Collaboration status among authors

VOSviewer is a bibliometric analysis software for constructing and visualizing bibliometric maps. It was co-developed by Nees Jan van Eck and Ludo Waltman of the Leiden University in The Netherlands [1], and it has unique advantages in clustering techniques based on co-occurrences. VOSviewer provides three types of map visualizations: network visualization, overlay visualization, and density visualization. VOSviewer was used in this study to analyze the collaboration status among authors, and the network visualization and overlay visualization of VOSviewer were employed. The network visualization could provide clusters of top authors in the field. This, together with the overlay visualization, could provide the distribution of timing of collaboration in each author cluster to understand their collaboration trends. Then, the directions of collaboration and research objectives of each author cluster could be obtained through reviewing the corresponding articles. When performing analysis using VOSviewer in this study, the minimum number of documents of an author was set to 20. As shown in Figure 4A, the article authors were divided into six large clusters, and Figure 4B shows the distribution of collaboration time among the authors.

B

A


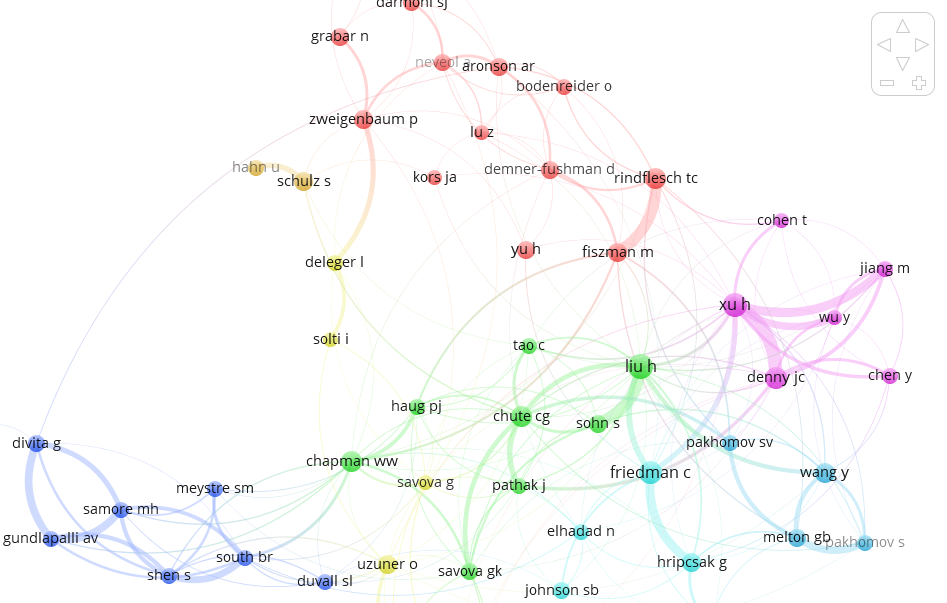

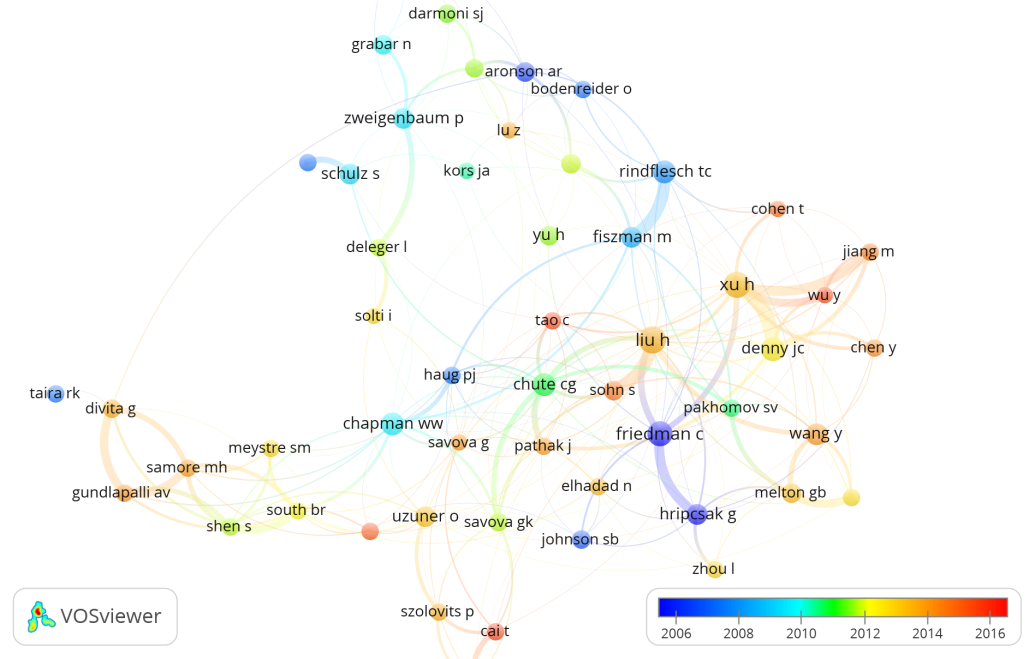


Figure 4 (A) Network visualization of author co-occurrences analyzed using VOSviewer. A circle represents an author, the size of the circle represents the importance, and the thickness of the link connecting the circles represents the relatedness of the connections. Circles with the same color belong to the same cluster. (B) Overlay visualization generated in VOSviewer. A color closer to blue represents an earlier time and closer to red represents a time closer to 2018.

## Keyword analysis

Analysis of keywords can indirectly reveal the hotspots and changing trends in research topics, which is critical for understanding the development of this field [2]. VOSviewer was used in this study to perform keyword analysis. The purpose of the analysis was to identify the most popular research hotspots in the field and to obtain the changing trends in keywords over time through the overlay visualization generated in VOSviewer. This could help researchers determine potential future research directions. During the statistical analysis, keywords were defined as words that were used more than 50 times in titles and abstracts in all publications. As shown in Figure 5A, a total of 327 keywords were identified, and the keywords were grouped as red, yellow, and blue. Based on these three categories, the relatedness among these keywords can be observed. For example, in the red category, patient (978 times), electronic health record (610 times), and electronic medical record (361 times) belong to the clinical NLP field; in the blue category, classifier (249 times), machine learning (215 times), support vector machine (164 times), and information extraction (150 times) belong to NLP research methods; and in the green category, language (449 times), phrase and word (395 times), ontology (345 times), terminology (267 times), and lexicon (106 times) belong to NLP research subjects. Next, the overlay visualization (Figure 5B) shows the trends in keyword changes as time progresses. In Figure 5B, blue indicates that the timing of appearance is earlier, and red indicates that the timing of appearance is later. The figure reveals that certain hotspots have developed in the field in recent years, including electronic health record (176 times in 2014), cancer (19 times in 2014), and machine learning (34 times in 2014). It is worth noting that social media in the red category appeared 22 times in 2016.

B

A


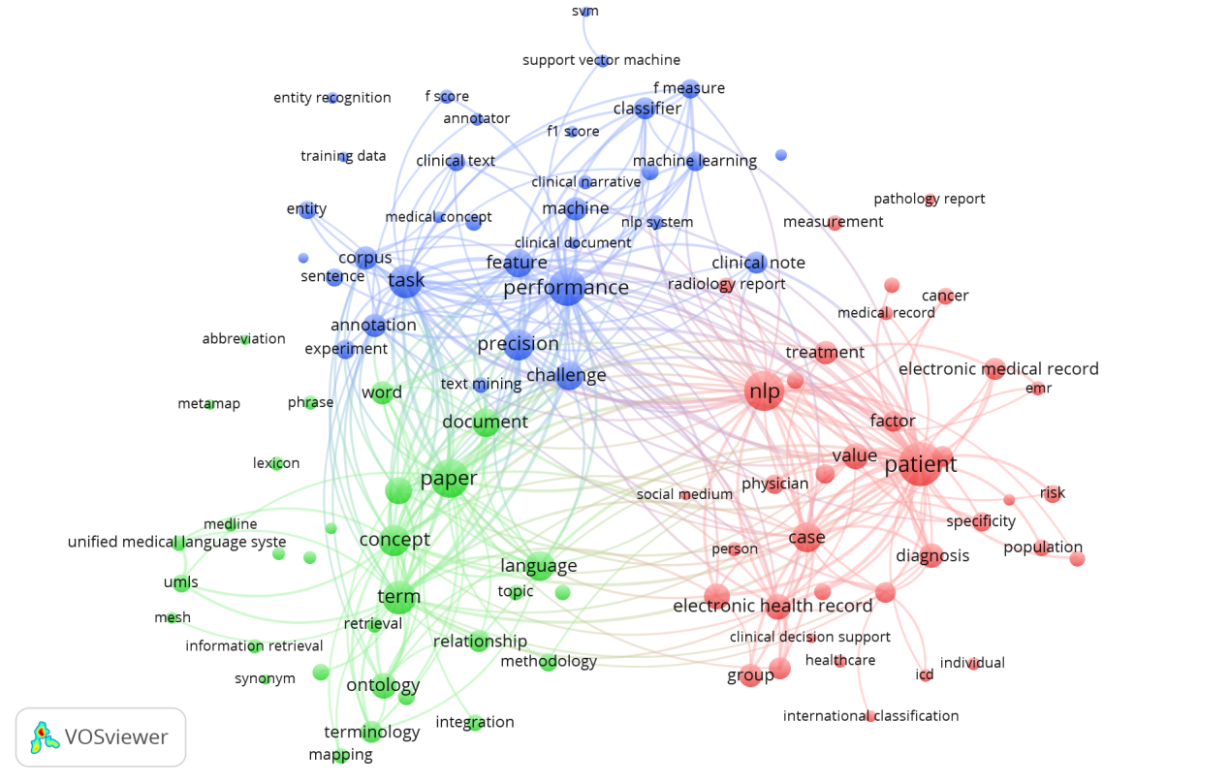


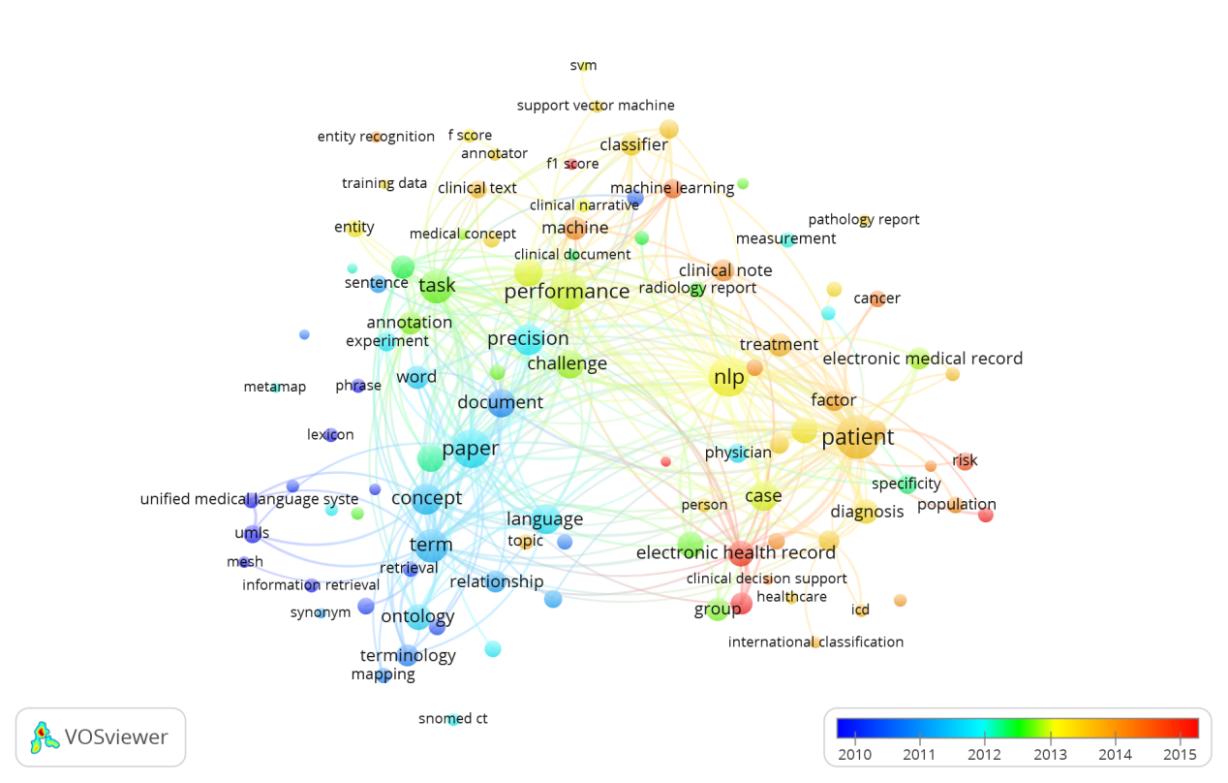


Figure 5 (A) Distribution of keywords. A circle represents an identified keyword, the size of the circle represents the importance, and the thickness of the link connecting the circles represents the relatedness of the connections among the keywords. Circles with the same color belong to the same cluster. (B) Changes in keywords over time. A color closer to blue represents an earlier time and closer to red represents a time closer to 2018.

# Discussion

## Analysis of collaboration status among authors and Analysis of Keywords

These two sections both used the VOSviewer tool to visualize and analyze the collaboration relationship among authors and research topics through keyword clustering repectively. Interested readers can view the detailed VOSviewer generated network diagrams and related discussion through the supplementary “Network diagrams and Analysis of keywords and collaboration among authors”

The VOSviewer tool was used for analysis in this study: First, Figure 4A is a network visualization, where a circle represents an author, the size of the circle represents the importance, and the thickness of the link between the circles represents the relatedness of the connections. Circles with the same color belong to the same cluster. The clustering algorithms of VOSviewer have been previously described [3, 4]. This type of coupling analysis of authors (co-authorship) can reveal the similarities and differences in the research topics conducted among researchers in the medical NLP field. Next, Figure 4B is an overlay visualization. Each node contains temporal information, and the color of the node is determined by the corresponding time. As show in Figure 4A, the collaboration status of the authors is distributed into 6 clusters. The authors within each cluster shared common research areas, as described in the following section.

(1) Purple: By combining the two figures, it can be seen that Hua Xu and Joshua C Denny had been collaborating frequently since 2010. Their research focus was on the extraction of information from electronic records, such as information on colonoscopy and drug treatment, and later on, the focus was on the identification of named entities and acronyms, such as “A study of active learning methods for named entity recognition in clinical text.” [5]. In addition, the articles co-authored by them often involve Min Jiang, Yukun Chen, and Yonghui Wu.

(2) Blue: There is a combination of three people in this cluster: Guy Divita, Matthew H. Samore, and Adi V. Gundlapalli. These three authors collaborated frequently around 2014, and the main research area was the identification and extraction of concepts from clinical cases. In particular, the three authors had also studied sexual trauma among American veterans [6,7]. In addition, some articles published by these three authors were co-authored by Brett R. South and Stéphane Meystre.

(3) Red: Thomas C. Rindflesch and Marcelo Fiszman collaborated mainly around 2010, with a primary focus on semantic abstraction and semantic predications, such as in “Adapting semantic natural language processing technology to address information overload in influenza epidemic management.” [8]

(4) Green: Hongfang Liu and Sunghwan Sohn co-authored several papers published during 2015–2018. Their main study area was NLP-assisted disease research, with a focus on asthma and peripheral pretrial disease [9-11]. They had also collaborated with others to study EHR. Furthermore, Christopher G. Chute and Hongfang Liu also had several collaborations, especially on cervical cancer [12].

(5) Light blue: Carol Friedman and George Hripcsak mainly collaborated around 2009. Their main research content was symptom detection and drug monitoring through examining electronic records, such as in “Active computerized pharmacovigilance using natural language processing statistics, and electronic health records: a feasibility study.” [13] Serguei V. Pakhomov and Genevieve B. Melton collaborated frequently around 2014, with a focus on semantics [14].

(6) Yellow: In this category, Louise Deleger and Imre Solti published several articles on how to prepare, establish, develop, and evaluate gold standards in clinical NLP between 2012 and 2014 [15-17], such as in “Building gold standard corpora for medical natural language processing tasks.” [18]

## Keyword analysis

The VOSviewer software tool divided the keywords into three categories through clustering. In the blue category, the top ranked keywords were classifier, machine learning, support vector machine, and information extraction. In addition, as shown in Figure 5B, the most prominent red circle in this category is the F1-score, which appeared 29 times in 2015 and is a recent hotspot in this category. The F1-score is a metric for classification problems and evaluation of retrieved results. Some NLP models or machine learning competitions on multiclass classification problems often use the F1-score as a means of final evaluation [15]. In 2015, which was also a year of exploding development in machine learning, companies such as Google, Microsoft, and Ford had all taken substantial actions in the field of machine learning. The top ranks in the red category were occupied by electronic health record, electronic medical record, and medication. It is worth noting that in this category, in addition to the common hotspots such as EMR, there is also a circle with a reddish color, indicating that it is a recent popular hotspot in this category: social media. Social media appeared 22 times in 2016. Through reviewing related articles, it was found that these articles obtained study data through social media, with the following two primary focuses: (1) pharmacovigilance, such as identifying adverse drug events and monitoring of prescription medication abuse [16,17]; (2) investigations on disease-related content, such as cancer care, or content related to diseases such as inflammatory bowel disease, arthritis, and mental illness [19]. The present study found that in research on social media, 20% of the articles obtained data through the Twitter platform, such as in “Enhancing seasonal influenza surveillance: topic analysis of widely used medicinal drugs using Twitter data” [20] and “Using Twitter to understand the human bowel disease community: exploratory analysis of key topics.” [21] In addition, articles related to social media were mainly published in the Journal of Medical Internet Research. The green category mainly included phrase, word, terminology, and lexicon, which were predominantly used as the subject of research and processing.

**Reference**

1. Van Eck, N.J., & Waltman, L. How to normalize cooccurrence data? An analysis of some well-known similarity measures[J]. Journal of the American Society for Information Science and Technology, 2009,60(8), 1635–1651.

2. Li T, Ho YS, Li CY . Bibliometric analysis on global Parkinson’s disease research trends during 1991-2006[J]. Neurosci Lett 441: 248-252.PMID:18582532

3. Noack A . Energy Models for Graph Clustering[J]. Journal of Graph Algorithms & Applications, 2008, 11(2):453-480.

4. Nees Jan Van Eck, Ludo Waltman. How to normalize cooccurrence data? An analysis of some well-known similarity measures[J]. Journal of the American Society for Information Science and Technology, 60(8), 1635–1651.

5. Yukun Chen, Thomas A. Lasko, et al. A Study of Active Learning Methods for Named Entity Recognition in Clinical Text[J]. Journal of Biomedical Informatics, 2015, 58:11-18.PMID:26385377

6. Gundlapalli A V , Brignone E , Divita G , et al. Using Structured and Unstructured Data to Refine Estimates of Military Sexual Trauma Status Among US Military Veterans[J]. Stud Health Technol Inform, 2017, 238:128-131.PMID:28679904

7. Divita G , Brignone E , Carter M E , et al. Extracting Sexual Trauma Mentions from Electronic Medical Notes Using Natural Language Processing[J]. Studies in Health Technology & Informatics, 2017, 245:351.PMID:29295114

8. Keselman A , Rosemblat G , Kilicoglu H , et al. Adapting Semantic Natural Language Processing Technology to Address Information Overload in Influenza Epidemic Management[J]. Journal of the American Society for Information Science and Technology, 2010, 61(12):2531-2543.PMID:24311971

9. Kaur H , Sohn S , Wi C I , et al. Automated chart review utilizing natural language processing algorithm for asthma predictive index[J]. BMC Pulmonary Medicine, 2018, 18(1):34.

10. Sohn S , Wang Y , Wi C I , et al. Clinical documentation variations and NLP system portability: a case study in asthma birth cohorts across institutions[J]. Journal of the American Medical Informatics Association, 25(3),2018,353-359.PMID:29439692

11. Afzal N , Sohn S , Abram S , et al. Identifying Peripheral Arterial Disease Cases Using Natural Language Processing of Clinical Notes[C]. In Proceedings of 3rd IEEE International Conference on Biomedical and Health Informatics (BHI 2016), pages 126-131. IEEE, 2016.

12. Wagholikar K B , Maclaughlin K L , Casey P M , et al. Automated Recommendation for Cervical Cancer Screening and Surveillance[J]. Cancer Informatics, 2014, 13(suppl 3):1-6.PMID:25368505

13. Wang X , Hripcsak G , Markatou M , et al. Active computerized pharmacovigilance using natural language processing, statistics, and electronic health records: a feasibility study.[J]. Journal of the American Medical Informatics Association, 2009, 16(3):328-337.PMID:19261932

14. Zhang R , Cairelli M J , Fiszman M , et al. Using semantic predications to uncover drug-drug interactions in clinical data[J]. Journal of Biomedical Informatics, 2014, 49(C):134-147.PMID:24448204

15. Hassanpour S , Langlotz C P , Amrhein T J , et al. Performance of a Machine Learning Classifier of Knee MRI Reports in Two Large Academic Radiology Practices: A Tool to Estimate Diagnostic Yield[J]. American Journal of Roentgenology, 2017, 208(4):750-753.PMID:28140627

16. Liu J , Zhao S , Zhang X . An ensemble method for extracting adverse drug events from social media[J]. Artificial Intelligence in Medicine, 2016, 70(9):62-76.PMID:27431037

17. Bousquet C , Dahamna B , Guilleminlanne S , et al. The Adverse Drug Reactions from Patient Reports in Social Media Project: Five Major Challenges to Overcome to Operationalize Analysis and Efficiently Support Pharmacovigilance Process[J]. Jmir Research Protocols, 2017, 6(9):e179.PMID:28935617

18. Deleger L , Li Q , Lingren T , et al. Building gold standard corpora for medical natural language processing tasks.[J]. AMIA. Annual Symposium proceedings / AMIA Symposium. AMIA Symposium, 2012, 2012:144-153.PMID:23304283

19. Conway M , Daniel O’Connor. Social Media, Big Data, and Mental Health: Current Advances and Ethical Implications[J]. Current Opinion in Psychology, 2016, 9:77-82.PMID:27042689

20. Kagashe I , Yan Z , Suheryani I . Enhancing Seasonal Influenza Surveillance: Topic Analysis of Widely Used Medicinal Drugs Using Twitter Data[J]. Journal of Medical Internet Research, 2017, 19(9):e315.PMID:28899847

21. Martín Pérez-Pérez, Gael Pérez-Rodríguez,et al. Using Twitter to Understand the Human Bowel Disease Community: Exploratory Analysis of Key Topics[J]. Journal of Medical Internet Research, 2019(Aug 13);21(8):e12610.PMID:31411142
